# Country differences in the diagnosis and management of coronary heart disease – a comparison between the US, the UK and Germany

**DOI:** 10.1186/1472-6963-8-198

**Published:** 2008-09-29

**Authors:** Olaf von dem Knesebeck, Markus Bönte, Johannes Siegrist, Lisa Marceau, Carol Link, Sara Arber, Ann Adams, John McKinlay

**Affiliations:** 1 Department of Medical Sociology, University Medical Center, Hamburg-Eppendorf, Germany; 2 Department of Medical Sociology, Heinrich-Heine-University, Duesseldorf, Germany; 3 New England Research Institutes, Boston, USA; 4 Department of Sociology, Centre for Research on Ageing and Gender, University of Surrey, UK; 5 Health Sciences Research Institute, Warwick Medical School, University of Warwick, UK

## Abstract

**Background:**

The way patients with coronary heart disease (CHD) are treated is partly determined by non-medical factors. There is a solid body of evidence that patient and physician characteristics influence doctors' management decisions. Relatively little is known about the role of structural issues in the decision making process. This study focuses on the question whether doctors' diagnostic and therapeutic decisions are influenced by the health care system in which they take place. This non-medical determinant of medical decision-making was investigated in an international research project in the US, the UK and Germany.

**Methods:**

Videotaped patients within an experimental study design were used. Experienced actors played the role of patients with symptoms of CHD. Several alternative versions were taped featuring the same script with patients of different sex, age and social status. The videotapes were shown to 384 randomly selected primary care physicians in the three countries under study. The sample was stratified on gender and duration of professional experience. Physicians were asked how they would diagnose and manage the patient after watching the video vignette using a questionnaire with standardised and open-ended questions.

**Results:**

Results show only small differences in decision making between British and American physicians in essential aspects of care. About 90% of the UK and US doctors identified CHD as one of the possible diagnoses. Further similarities were found in test ordering and lifestyle advice. Some differences between the US and UK were found in the certainty of the diagnoses, prescribed medications and referral behaviour. There are numerous significant differences between Germany and the other two countries. German physicians would ask fewer questions, they would order fewer tests, prescribe fewer medications and give less lifestyle advice.

**Conclusion:**

Although all physicians in the three countries under study were presented exactly the same patient, some disparities in the diagnostic and patient management decisions were evident. Since other possible influences on doctors treatment decisions are controlled within the experimental design, characteristics of the health care system seem to be a crucial factor within the decision making process.

## Background

There is an increasing interest in health care variations between different national systems. Geographic variations in health care depending on where the patient lives and the system in which care is received have been shown in a couple of studies, including studies dealing with the management of coronary heart disease (CHD) [1-5]. One possible explanation for such international variations in health care lies in different systems of health care financing and reimbursement that may cause different priorities in patient management [6]. According to the 'supplier-induced demand' thesis [7-9] physicians have the ability to generate demand and thus, differences in financial incentives for providing services could lead to variations in health care in different countries. Evidence-based medicine has emerged as an international health care paradigm which promotes the use of tools like disease management programs and clinical guidelines, to influence provider decision making, improve the quality of care and reduce both national and eventually international variations [5]. However, while disease management programs for CHD patients are well established in the US and the UK, countries like Germany have just recently started to build such managed care structures.

Against this background comparative findings from an international research project on clinical decision making in patients with symptoms of CHD are presented in this paper. We have chosen three countries, each with a different organisation and financing of health care (a largely private insurance-based health care system in the US, a National Health Service government-supported, tax-based system in the UK, and a system characterized by decentralized care administered by social security agencies in Germany). The aim of the study was to simultaneously measure the effect of country/the health care system, patient attributes, and physician characteristics on disparities in clinical decision making of primary care providers. This paper focuses on differences between the countries under study.

## Methods

### Experimental study design

A factorial experiment with a videotaped patient consultation was conducted [10-14]. Due to the experimental approach relevant patient (age, gender, social status, race) and physician factors (experience, gender) are controlled by the study design. Professional actors played the role of patients with symptoms of CHD. Patients in the video-vignette presented several symptoms that are typical for CHD including chest pressure; pressure worsened with exertion, stress and eating; relief after resting; discomfort for more than three months; pain through the back between the shoulder blades; and elevated blood pressure. Additionally a non-verbal cue was incorporated, demonstrated by the 'Levine fist' (clenched fist to the sternum) [15,16]. Tape-recorded role-playing sessions were conducted with experienced clinical advisors. From these tapes one script was developed that was used for all videotapes presented to physicians with appropriate language (British English vs. American English) modifications. The portrayed patients in the US had American accents, while the very same in the UK had English accents. For the German part of the study the US-videotapes were dubbed by a professional speaker. To this end, scripts were translated into German and then back into English to identify discrepancies between original and back-translation. This forward-backward process was repeated until satisfactory agreement was attained. To ensure clinical accuracy and comparability with the other two countries the speaker was asked to adhere to the translated script, instead of giving priority to synchrony. Moreover, care was taken to construct a culturally neutral medical practice setting for the filming. The patient in the videotapes was portrayed as having consulted this doctor before and having recently returned from vacation/holiday. The videos were presented to primary care physicians in their own practice rooms. Actors on the tapes differ according to their age (55 vs. 75 years), gender (male vs. female) and social status (low vs. high depicted by their current/former occupation of a janitor/cleaner or school teacher) in order to mirror respective patient characteristics. In addition race (black vs. white) was varied in the US and the UK. As black patients in Germany are very rare, this characteristic was not varied in the videos in Germany. One CHD-video was presented to each physician. After viewing the tapes the physicians were asked a range of questions concerning patient management by using a questionnaire with both standardised and open-ended questions. The physicians were asked what questions they would ask, to name the most likely diagnoses, what their certainty levels were, which test(s) they would order, which medication(s) they would prescribe, and what lifestyle recommendation(s) they would make if they saw the patient from the video in their everyday clinical practice. In terms of information seeking behaviour, the question was whether the physicians would ask the patient any additional questions (yes/no) and if so, what kind of question(s) they would ask (open ended). In terms of diagnostic decision making, doctors were asked what they thought was going on with the patient (open ended) and how certain they were with their diagnostic decision (from 0 to 100). Moreover, physicians were asked whether they would order any tests for this patient (yes/no) and to name the tests they would order (open ended). In terms of therapy, we wanted to know if doctors would prescribe or recommend any medication (yes/no), if they would refer the patient to another health care professional (yes/no) and when they would like to see the patient again (in days). Finally, physicians were asked if they would recommend any lifestyle advice or behaviour change (yes/no) and if so, what kind of advice they would give (open ended).

### Physician sample

The study was conducted in the US (Massachusetts), in the UK (the West Midlands, Southeast London and Surrey) and in Germany (North Rhine-Westphalia). Physicians were randomly selected from lists provided by local health care organizations. Selection was made within four strata, defined by combinations of the physicians' gender and length of clinical experience. In order to get a clear separation by level of experience only physicians with less than 5 years or more than 15 years clinical experience were considered eligible for selection. Moreover, physicians had to be internists or family practitioners in the US and in Germany or general practitioners in the UK. For a clear separation by country they had to be trained at a medical school in their respective countries. Altogether 384 interviews were conducted in the three countries (128 in each country). For a sample size of 384 physicians the design has 80% power to detect absolute differences of 14.5 percent between two groups. For example, if 14.5% of physicians would refer a patient to a cardiologist/specialist facility in one country and 29% of physicians would refer a patient to a cardiologist/specialist facility in another (a difference of 14.5%) then we would expect to find a significant difference (at alpha = 0.05) 80 percent of the time. Randomly selected physicians were sent a letter of invitation. Thereafter, these physicians were screened over the phone so that eligible doctors could be identified. An appointment was scheduled for the personal one hour semi-structured interview in the physicians practice. Data were collected in 2001/2002 in UK and the US, and in 2004/2005 in Germany. Participating doctors received a modest financial acknowledgement. The response rates were 64.9% in the US, 59.6% in the UK, and 65% in Germany. Informed consent was signed by all participating physicians. This consent form was approved as required by the US Institutional Review Board and conformed to requirements of UK and German ethics committees. The overall study was approved by the New England Research Institutes Institutional Review Board.

### External validity

Since videotaped patients were used, physicians may behave differently under experimental conditions compared with real patients in their everyday clinical work. To address this issue several steps were taken to foster the external validity of the study design. High effort was devoted to increase the clinical realism of the vignettes. Due to the conducted role-play sessions, the scripts are based on clinical experience. At the filming, experienced clinicians were present on the set and only professional actors were engaged. At the beginning of each interview during the fieldwork doctors were specifically asked how typical the patient on the videotape was, compared to patients they encounter in everyday practice. 90.6% considered them either very typical or reasonably typical in the US, 91.4% in the UK and 81.3% in Germany. Additionally, all physicians viewed the tapes in the context of their own practice during normal practice hours so that it was likely they encountered real patients before and after they participated in the study. Prior to the interview, all physicians were instructed to view the patient as one of their own patients, and to respond as they would typically respond in their own practice.

### Statistical methods

Analysis of variance was used to assess the country effect on doctors' diagnostic and therapeutic decisions. Given the balanced factorial design, the main effect due to country is orthogonal to (independent of) the main effects due to physicians and patients factors. Tukey's studentized range multiple comparisons were performed to test statistical significance of differences between the three countries. Precise p-values are reported in the tables. P < 0.05 was considered to be an indication that the differences noted were statistically significant.

## Results

### Information seeking behaviour

Table 1 summarizes major differences between randomly sampled physicians in the US, the UK and in Germany in their information seeking behaviour. After the physicians saw the videotaped patient we wanted to know if they would ask the patient any additional questions. The average total number of questions is highest in the US (8.7), followed by the UK (5.5) and Germany (3.1). Several significant differences regarding topics raised also exist between the three countries under study. 83% of the US physicians would like to know more about the medical history, more than in the UK (62%) and in Germany (39%). Questioning related to pain differs significantly between Germany (32%) and the other two countries (US 62%, UK 57%). All three countries differ significantly with respect to questions referring to the patient's general health status (US 83% vs. UK 62% vs. Germany 30%). In terms of questions about smoking and alcohol consumption, US and UK physicians do not differ significantly, however in Germany, physicians' question behaviour is significantly different. Only 20% of the German GPs would ask about smoking (vs. US 57% and UK 48%) and only 8% would ask about alcohol consumption (vs. US 34% and UK 31%).

**Table 1:** Total numbers of additional questions and percentages of physicians who would ask the patient different types of additional questions by country

		***Countries***			
	***p country***	***Germany***	***UK***	***US***	***Tukey's Multiple Comparison***

Number of questions (means)	< .0001	3.1	5.5	8.7	US > UK > Germany
Questions about (%)					
pathology	.3879	69	77	71	UK = US = Germany
medical history	< .0001	39	62	83	US > UK > Germany
pain	< .0001	32	57	62	US = UK > Germany
smoking	< .0001	20	48	57	US = UK > Germany
alcohol	< .0001	8	31	34	US = UK > Germany
psychological state	.0226	20	33	35	US = UK > Germany
social	.0901	17	14	25	US = Germany = UK
general	< .0001	30	62	83	US > UK > Germany

### Diagnosis

Although all physicians in the three countries encountered exactly the same patient on the videotape, there are country differences in the percentage of medical practitioners who mentioned coronary heart disease as a possible diagnosis. Table 2 shows that 95% of the US, 88% of the UK and 74% of the German physicians mentioned CHD when they were asked what they think is going on with the patient. Only Germany significantly differs from the other countries in this regard. After mentioning CHD as a possible diagnosis, physicians were asked how certain on a scale from 0 to 100 they are with their diagnosis (for physicians who did not give a CHD diagnosis, their certainty was set to 0). The level of certainty significantly differs between the US (58%) and the two other countries (UK 46% and Germany 39%). There is no significant difference in the percentage of general practitioners who would order a test to diagnose a possible CHD between the US (89%) and the UK (80%), but again Germany (39%) differs significantly from the other countries. Taking a look at the total number of tests that were ordered to detect CHD, the same results can be found. Germany significantly differs (0.9 tests) from the US (3.2 tests) and the UK (3.5 tests).

**Table 2:** Coronary heart disease (CHD) diagnosis, certainty of diagnosis and test ordering for CHD by country

		**Countries**			
	***p country***	***Germany***	***UK***	***US***	***Tukey's Multiple Comparison***

CHD diagnosis (%)	< .0001	74	88	95	US = UK > Germany
Certainty of CHD diagnosis (0–100)	< .0001	39	46	58	US > UK = Germany
Test for CHD (%)	< .0001	39	80	89	US = UK > Germany
Number of tests for CHD (means)	< .0001	0.9	3.5	3.2	UK = US > Germany

### Therapy

Results on therapy decisions are presented in Table 3. The prescribing behaviour differs significantly in all three countries. 67% of the US general practitioners would prescribe or recommend a medication before having the results of any test ordered. In the UK 48% would do the same, whilst in Germany only 17% of the physicians would prescribe a drug. In terms of referrals to a cardiologist there are significant differences between the UK (31%) and the other two countries. Referral to another medical specialist significantly differs amongst all countries (US 5% vs. UK 30% vs. Germany 52%). Although the time to the next appointment does not significantly vary between the US (11.3 days) and the UK (13.1 days), German physicians would like to see the patient again significantly sooner (5.7 days).

**Table 3:** Therapy decisions by country

		**Countries**			
	***p country***	***Germany***	***UK***	***US***	***Tukey's Multiple Comparison***

Prescribing appropriate CHD medication (%)	< .0001	17	48	67	US > UK > Germany
Referral to a cardiologist or specialist facility (%)	< .0001	19	31	10	UK > Germany = US
Referral to other medical professional (%)	< .0001	52	30	5	Germany > UK > US
Time to next appointment (days, means))	< .0001	5.7	13.1	11.3	UK = US > Germany

### Lifestyle advice

The physicians were asked whether they would give any lifestyle advice. General practitioners in the US (2.8 pieces) and the UK (2.9 pieces) do not differ in the average number of pieces of advice, but the German GPs would give significantly less advice (1.9 pieces) (Table 4). Country differences can additionally be found by the kind of advice that was given. Large and significant differences are evident for the recommendation to stop smoking (US 32% vs. UK 55% vs. Germany 9%). The suggestion to reduce alcohol consumption was given by 18% of the physicians in the US and 36% in the UK. Only 10% of the German GPs would raise this topic, but the difference to the UK and the US failed significance. In addition minor non-significant differences in the advice for weight reduction, exercise and diet were found.

**Table 4:** Total Numbers and percentages of physicians who give different types of lifestyle advice and recommendations by country

		**Countries**			
	***p country***	***Germany***	***UK***	***US***	***Tukey's Multiple Comparison***

Total numbers of advice (means)	< .0001	1.9	2.9	2.8	UK = US > Germany
Advice about (%)					
diet	.7507	51	53	55	US = UK = Germany
smoking	< .0001	9	55	32	UK > US > Germany
alcohol	< .0001	10	36	18	UK > US = Germany
relaxation	.1873	9	11	5	UK = Germany = US
exercise	.7643	7	9	6	UK = Germany = US
weight	.0824	3	8	2	UK = Germany = US

## Discussion

This paper focuses on between-country differences in clinical decision making in CHD. Doctors' diagnostic and management decisions were compared in three countries (US, UK and Germany) with different health insurance systems. To analyse the country effect on doctors' decisions a factorial experiment with video vignettes was conducted. Working with vignettes is a sophisticated and valid method for measuring the quality of care provided by primary care physicians [17]. This design was successfully used in previous studies to estimate the un-confounded effects of non-medical factors in clinical decision making [10,18,19]. While this rigorous experimental study permits excellent internal validity, external validity remains a threat. Substantial effort was devoted to produce the video vignettes to be as realistic as possible. However, patient management decisions result from the interaction between physician and patient and from clinical examination. These issues could not be adequately addressed in a videotape-based experiment. Furthermore, doctors may have viewed the interview as a test situation. This could possibly bias the answers in the direction of social acceptability. To avoid this, the doctors were specifically told that the interview is not a test, and that we are interested in their daily work and not in textbook answers.

While there are some methodological limitations, the design has considerable strengths. The factorial experiment allows the estimation of independent and un-confounded country effects as it simultaneously controls for different types of patient (age, gender, social status, and race) and provider influences (gender and length of clinical experience) on clinical decision making. Although there are more physician and patient characteristics, these are considered important for the decision making process. In addition, the experimental approach with videotaped patients offers the possibility to integrate non-verbal signs such as the 'Levine fist'. To enhance generalizability doctors were randomly selected.

Even though all patients reported exactly the same symptoms, results indicate differences between countries, especially between Germany on the one hand and the US and the UK on the other. Minor non-consistent differences were found in patient management between British and American physicians (see also [5]): British doctors gave lifestyle recommendations regarding alcohol and smoking more often and referred their patients to a cardiologist or other medical specialist more frequently. American doctors were more certain about a CHD diagnosis, they would request more additional information from the patient, and they provided more prescriptions appropriate to CHD. Physicians in Germany showed a significantly different pattern of behaviour. They asked fewer additional questions, diagnosed CHD less often, and were also less certain with their diagnosis compared to UK and US doctors. Among German physicians, a minor proportion would order a CHD specific test and if so, they would order fewer tests. Medications were prescribed to a lesser extent, but patients would see the doctor again sooner. Finally the total number of pieces of lifestyle advice given to the patient and the kind of advice significantly differs from the UK and US. Overall, German physicians would be less active in terms of diagnostic and management strategies. The reasons for this discrepancy may be structural.

In our study physicians were asked how much time they do have for a patient consultation (e.g., for a routine patient consultation, American physicians are allocated on average 18 minutes, British physicians are allocated 10 minutes, and German physicians are allocated 5.5 minutes). Generally, German physicians have the least time for patient consultations compared with UK and especially with US doctors. At the same time, German physicians would like to see the patient again much sooner. Thus, physicians in Germany seem to have the smallest time allocation for a single consultation while they would see the patients in smaller intervals. These different time restrictions might cause varying treatment strategies. Furthermore, there is a relationship between organizational structure and clinical performance [20]. Differing structures of health care financing and reimbursement are likely to influence clinical decision making. Thus, characteristics of the health care system in each country might be one explanation for the observed country differences.

Although we consider these structural reasons most important, other explanations can't be ruled out. First, there might be cultural reasons for our results indicating that German physicians are less active in terms of diagnostic and management strategies than their US and UK colleagues. For example, physicians' behaviour may reflect different patient expectations. Secondly, there are possible methodological reasons for the different physicians' behaviour in Germany, as data were collected 3 years later than in the other two countries and vignettes in Germany were dubbed. The slightly different way that the videotape was made may have altered physicians' perception of the patient in Germany. However, more than 80% of the German physicians considered the patient on the videotape either very typical or reasonably typical compared to patients they encounter in everyday practice (in the UK and the US this rate was about 90%).

## Conclusion

Results indicate country differences in clinical decision making related to CHD, especially between Germany on the one hand and the US and the UK on the other. Particularly structures of health care financing and the state of evidence-based care programs can explain the observed country differences. In this regard, incentives for different diagnostic and therapeutic procedures can be seen as predictors of clinical decisions.

## Competing interests

The authors declare that they have no competing interests.

## Authors' contributions

OvdK and MB were responsible for interpreting the data and writing the manuscript. CL was responsible for analysing the data, contributed to writing and provided feedback on drafts. JS, LM, SA, AA and JM contributed to writing the article and provided feedback on drafts. All authors were involved in conducting the study (Grant No. AG 16747 from the National Institute on Aging, NIH).

## Pre-publication history

The pre-publication history for this paper can be accessed here:


